# A Retrospective Study of Replacement Resorption and Its Risk Factors After Replantation of Avulsed Young Permanent Teeth

**DOI:** 10.1002/cre2.70254

**Published:** 2025-12-10

**Authors:** Gong Min, Qi Jianyan, Cai Chenxing, Gao Suyu

**Affiliations:** ^1^ Department of Pediatric Dentistry The Affiliated Stomatological Hospital of Nanjing Medical University Nanjing China; ^2^ State Key Laboratory Cultivation Base of Research Prevention and Treatment for Oral Diseases Nanjing China; ^3^ Jiangsu Province Engineering Research Center of Stomatological Translational Medicine Nanjing China

**Keywords:** replacement resorption, tooth ankylosis, tooth avulsion, tooth replantation, young permanent teeth

## Abstract

**Objective:**

A retrospective study was conducted on 30 cases of replacement resorption after replantation of avulsed young permanent teeth with clinical observation period of more than 2 years, to provide reference for clinical treatment and prevention.

**Methods:**

A retrospective study was carried out on 30 cases replacement resorption after replantation of avulsed young permanent teeth with replacement resorption. The clinical and imaging data during posttraumatic follow‐up were recorded and analyzed, including trauma age, gender, traumatic tooth position, root development stage, time of tooth separation, storage media, fixed time, and clinical examination.

**Results:**

Among the 30 cases, the fastest replacement resorption occurred in the first month after trauma (1 case), and the slowest occurred in the 24th month (1 case). The incidence of replacement resorption was 26.7% at month 3, 70% at month 6, 90% at month 9, and 96.7% at month 12. Fifteen patients showed progressive ankylosis, the degree of ankylosis gradually increased. Analysis and screening of risk factors found that the extraoral time was statistically significant.

**Conclusion:**

The study shows that the longer the time in vitro, the more likely progressive tooth sinking will occur, resulting in tooth loss. Therefore, it is more important to popularize the emergency treatment of mean avulsion. How to correctly deal with prolapse teeth, how to optimize the emergency trauma channel, reduce the occurrence of delayed replantation, and prolong the life of replantation teeth will be the focus of the future work of pediatric stomatologists.

## 1 Introduction

Dental trauma poses a substantial public health challenge, particularly within the pediatric and adolescent populations, with the avulsion of permanent teeth ranking among the most critical forms of dental injury. The clinical management of avulsed young permanent teeth presents distinct challenges, given that these teeth are at a pivotal stage of root development and are particularly vulnerable to complications such as replacement resorption (RR) (Caeiro‐Villasenín et al. 2022). RR, a pathological process marked by the gradual replacement of dental root tissues with alveolar bone, often culminates in tooth ankylosis and infraposition (Huang et al. 2024). These sequelae not only cause significant functional and aesthetic impairments but also exert a profound influence on a child's overall quality of life (Lin et al. 2022).

The incidence of dental trauma reaches its zenith during the ages of 7–9 years, a period marked by mixed dentition when children exhibit heightened physical activity but have yet to fully develop protective reflexes (Jones 2020). Epidemiological investigations have consistently demonstrated that avulsion injuries constitute approximately 1%–16% of all traumatic dental injuries affecting young permanent teeth, with the maxillary central incisors being the most commonly involved. The prognosis of replanted avulsed teeth hinges on a multitude of factors, encompassing the duration the tooth remains outside the oral cavity (extraoral time), the choice of storage medium, the stage of root development, and the technical finesse employed during the replantation procedure (Lam 2016). Among these determinants, the extraoral time stands out as the most pivotal factor, with teeth replanted within a 15‐min window exhibiting the greatest propensity for periodontal ligament healing. Conversely, teeth subjected to dry storage exceeding 60 min almost inevitably progress to RR.

RR refers to the process in which, following root resorption, the root is gradually replaced by bone tissue, leading to ankylosis of the tooth (Warrilow et al. 2021). It often occurs secondary to delayed replantation after avulsion injury (when the tooth has been out of the socket for more than 1 h) or severe intrusive injuries (with intrusion depth greater than 6 mm). Unlike surface inflammatory resorption, RR is a progressive process that cannot be halted. The root gradually fuses pathologically with the alveolar bone, and the tooth crown may subside. The rate of subsidence and ankylosis is closely related to the patient's age (Abbott and Lin 2022). In adult patients, the rate of RR can be very slow, with the entire process potentially lasting over a decade or even several decades. However, for young permanent teeth in the growth and development phase, due to the high basal metabolic rate in children, RR leads to cessation of alveolar bone growth, causing subsidence and ankylosis at a faster rate. Fouad et al. (2020) found that after the diagnosis of RR, the entire root is usually resorbed within 3–7 years, resulting in the loss of the tooth crown.

Malmgren and Malmgren (2002) et al. classified the degree of ankylosis and subsidence of traumatized teeth into four grades based on the extent of subsidence: Grade I (mild), with subsidence less than 1/8 of the crown height; Grade II (moderate), with subsidence greater than 1/8 but less than 1/4 of the crown height; Grade III (severe), with subsidence greater than 1/4 but less than 1/2 of the crown height; and Grade IV (very severe), with subsidence greater than 1/2 of the crown height. Currently, there are no ideal preventive measures to halt the occurrence and progression of RR. Decoronation is currently considered the best treatment method for RR in young permanent teeth (Einy et al. 2020). There is also a significant lack of long‐term clinical data on the observation of RR following trauma.

This study conducted a retrospective analysis of 30 clinical cases of RR in young permanent teeth that had undergone replantation after complete avulsion, with a clinical observation period exceeding 2 years, aiming to provide reference materials for clinical treatment and prevention.

## 2 Materials and Methods

### 2.1 Study Design and Patient Selection

This retrospective cohort study analyzed clinical data from 30 patients who underwent replantation of avulsed young permanent teeth at the Department of Pediatric Dentistry, Affiliated Stomatological Hospital of Nanjing Medical University, between 2019 and 2020. The inclusion criteria were as follows: Single‐tooth avulsion and replantation of a maxillary central incisor (with the contralateral tooth serving as a control). Minimum follow‐up period of 24 months, with complete clinical and radiographic documentation at scheduled intervals (1, 3, 6, 9, 12, 15, 18, 21, and 24 months post‐trauma).

Diagnosis of RR based on at least one of the following clinical or radiographic criteria: Loss of physiological tooth mobility; high‐pitched metallic tone on percussion; progressive infraposition of the tooth; radiographic evidence of periodontal ligament space obliteration, or replacement of root structure by bone.

Exclusion criteria comprised: Patients lost to follow‐up during the observation period; cases with secondary trauma or orthodontic intervention during the study; teeth with pre‐existing developmental anomalies or prior endodontic treatment.

### 2.2 Data Collection and Variables

Demographic and clinical parameters were systematically extracted from medical records, including patient characteristics: Age at injury, sex, and compliance with follow‐up.

Tooth‐related factors: Root development stage (classified by Nolla's stages: very immature [Nolla 5–8, open apex] vs. immature [Nolla 9, nearly complete root length with wide apex]); extraoral time (recorded in hours, categorized as ≤ 1 h, 1–3 h, or > 3 h); storage medium (favorable: milk, saline, or saliva; unfavorable: dry storage or wrapped in paper); treatment variables: splinting duration (≤ 2 weeks vs. > 2 weeks); adjunctive procedures (e.g., root canal therapy, apexification, or regenerative endodontics).

### 2.3 Radiographic Assessment

Serial radiographic images (periapical radiographs, panoramic radiographs, or CBCT scans) were evaluated by two calibrated examiners to assess.

### 2.4 RR Progression

Index for infraposition of ankylosed incisors. The homologous maxillary incisors with healthy periodontal ligaments are used as reference teeth. Degree I. Minimal, < 1/8 of the crown height. Degree II. Moderate, ≥ 1/8 but, < 1/4 of the crown height. Degree III. Severe, ≥ 1/4 but < 1/2 of the crown height. Degree IV. Extreme, ≥ 1/2 of the crown height.

### 2.5 Statistical Analysis

Data were analyzed using SPSS 23.0 (IBM Corp.). Descriptive statistics summarized demographic and clinical variables. The chi‐square or Fisher's exact test compared categorical variables (e.g., storage medium vs. RR progression). Logistic regression identified risk factors for progressive infraposition (dependent variable: stable [Grade I maintained] vs. progressive [≥Grade II advancement]). Inter‐examiner reliability was assessed via Cohen's kappa (*κ* > 0.80). A *p*‐value < 0.05 denoted statistical significance.

### 2.6 Ethical Considerations

The study protocol was approved by the Institutional Review Board of Nanjing Medical University (approval no.: PJ2019‐120‐001). Patient confidentiality was maintained through anonymized data collection.

**Table 1:** Basic data statistics of cases (age, time out of socket, and fixation time).

	N	Min.	Max.	Mean	Std. Dev.
Age (years)	30	7	15	9.50	1.852
Time out of socket (hours)	30	2	48	6.17	9.570
Fixation time (weeks)	30	2	4	3.07	0.980

**Table 2:** Basic data statistics of cases (gender, root development stage, and preservation medium).

		Frequency	Percentage
Gender	Male	17	56.7
	Female	13	43.3
Root development stage	Very immature	8	26.7
	Immature	22	73.3
Preservation medium	Favorable	19	63.3
	Unfavorable	11	36.7

## 3 Results

### 3.1 Basic Case Data Statistics

A total of 30 children were included in the statistical analysis, comprising 17 males and 13 females, with an average age of trauma at 9.50 ± 1.852 years (Tables 1 and 2). Among them, 8 teeth had open root apices (Nolla stages 5‐8), indicating very immature root development, while 22 teeth had thickened root apices (Nolla stage 9), suggesting root development was nearly complete. The shortest time the teeth were out of the socket was 2 h, and the longest was 48 h, with an average of 6.17 h. Nineteen teeth were preserved in a favorable medium, while 11 were preserved in an unfavorable medium. The fixation time ranged from 2 to 4 weeks, with an average of 3.07 weeks.

### 3.2 Follow‐Up Observation of Replacement Resorption

#### 3.2.1 Onset Time of Replacement Resorption

Through follow‐up observation, the fastest occurrence of RR was observed in the first month after trauma (1 case), and the slowest occurred in the 24th month (1 case). The incidence rates of RR were 26.7% at 3 months, 70% at 6 months, 90% at 9 months, and 96.7% at 12 months.

##### 3.2.1.1 Degree of Replacement Resorption

**Table 3:** Degree of replacement resorption during the follow‐up period.

	1 Month	3 Month	6 Month	9 Month	12 Month	15 Month	18 Month	21 Month	24 Month
No infraposition	29	22	9	3	1	1	1	1	
Degree I	1	7	18	24	26	24	22	18	15
Degree II		1	3	3	2	3	5	9	10
Degree III					1	2	2	2	3
Degree IV									2
Proportion	3.3%	26.7%	70%	90%	96.7%	96.7%	96.7%	96.7%	100%

Over the 2‐year observation period, none of the 30 patients experienced tooth loss (Table 3). Fifteen patients consistently showed mild infraposition without significant acceleration. Fifteen patients exhibited progressive infraposition and root RR, with varying speeds and degrees of resorption. By the 24th month of follow‐up, all patients showed RR, with 15 cases of mild infraposition, 10 cases of moderate infraposition, 2 cases of extreme infraposition (infraposition greater than half the crown height), and 3 cases of severe infraposition.

##### 3.2.1.2 Speed of Replacement Resorption

Among the 30 patients, 15 showed stable infraposition (not exceeding I degree) over the 2‐year follow‐up period, while the other 15 exhibited progressive infraposition. A multivariate logistic regression analysis was conducted, dividing the sample into stable and progressive infraposition groups, and found that the time out of the socket was statistically significant.

**Figure 1:**
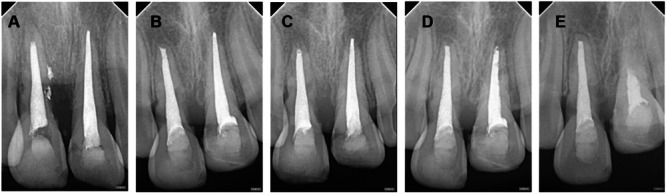
Case 1 of replacement resorption—extreme infraposition. A–E: Male, aged 10. Tooth 21 (with nearly complete root development) was replanted after 6 h out of the socket and showed extreme infraposition at the 21st month. A: 1 month after trauma, physiological mobility disappeared, and the periodontal membrane space was blurred. Infraposition was I degree (mild), less than 1/8 of the crown height. B: 6 months after trauma, progressive infraposition was observed. C: 9 months after trauma, infraposition was II degree. D: 15 months after trauma, infraposition was III degree. E: 21 months after trauma, infraposition was IV degree.

**Figure 2:**
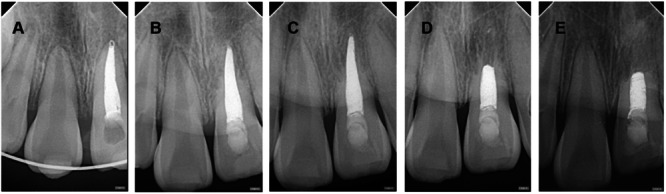
Case 2 of replacement resorption—replacement resorption began at 6 months after trauma. A–E: Male, aged 8. Tooth 21 was replanted after 2 h out of the socket and followed up. A: 1 month after trauma, apexification had been performed, and the periodontal membrane space was acceptable. B: 6 months after trauma, 1/3 of the root apex showed loss of the periodontal membrane space, indicating obvious replacement resorption. Infraposition was I degree (mild), less than 1/8 of the crown height. C: 12 months after trauma, 21 maintained mild infraposition. D: 15 months after trauma, the degree of infraposition remained I degree, with 1/2 of the root apex already replaced by bone tissue. E: 21 months after trauma, 1/2 of the root apex pulp cavity had disappeared, and infraposition was II degree.

**Figure 3:**
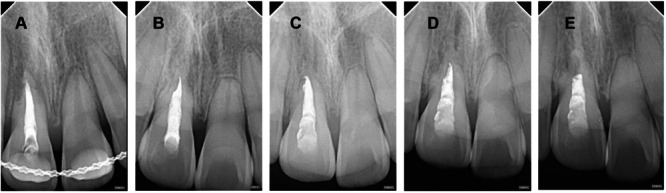
Case 3 of replacement resorption—infraposition, inflammatory resorption, replacement resorption. A–E: Female, aged 10. Tooth 11 was replanted after 2 h out of the socket and followed up. A: 1 month after trauma, apexification had been performed, showing inflammatory external resorption at 1/3 of the root apex. B: 6 months after trauma, 1/3 of the root apex showed loss of the periodontal membrane space, with adhesion between the alveolar bone and cementum, and 11 showed I‐degree infraposition. C, D: From 9 to 24 months after trauma, 11 maintained II‐degree infraposition, with continuous replacement resorption at 1/3 of the root apex and continuous replacement of alveolar bone for cementum. At 24 months after trauma, the distal root canal wall at 1/3 of the root apex was incomplete (E).

**Figure 4:**
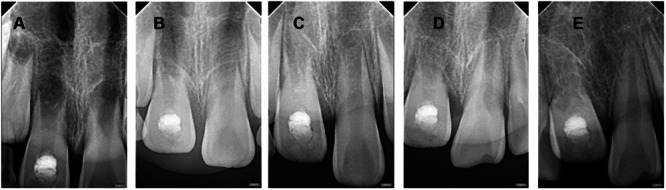
Case 4 of replacement resorption—stagnated root development, infraposition, replacement resorption after replantation of very immature permanent teeth. A–E: Female, aged 8. Tooth 11 was replanted after 5 h out of the socket and followed up. A: 1 month after trauma, pulp revascularization had been performed, with a trumpet‐shaped root apex. B: 3 months after trauma, the root of 21 developed normally. 11 showed II‐degree infraposition, with increased image density in the root canal and visible trabecular bone crawling into the root canal. C: 9 months after trauma, the root of 21 developed 2/3. 11 continued to infrapose to III degree. D: 15 months after trauma, 11 showed III‐degree infraposition, with most of the root replaced by alveolar bone. E: 21 months after trauma, the root of 21 was nearly complete. 11 continued to infrapose to IV degree, with complete replacement resorption of the root up to the cervical region.

**Figure 5:**
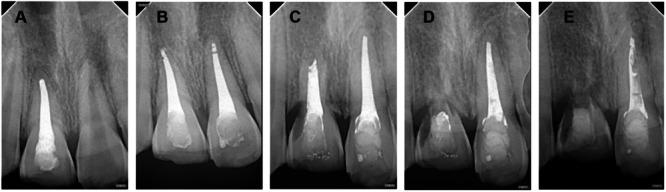
Case 5 of replacement resorption—replacement resorption to inflammatory resorption on the root cervix. A–E: Male, aged 10. Tooth 11 was replanted after 2 h out of the socket and followed up. A: 3 months after trauma, apexification had been performed, with 11 showing I‐degree infraposition. B: 9 months after trauma, 11 showed I‐degree infraposition. C: 12 months after trauma, 11 showed II‐degree infraposition and root replacement resorption. D, E: From 15 to 21 months after trauma, 11 showed III‐degree infraposition and progressive replacement resorption up to the cervical region, with disappearance of the root canal morphology.

Imaging tracking analysis of RR (selected root apex films with significant changes during the 2‐year follow‐up period) (Figures 1, 2, 3, 4, 5).

## 4 Discussion

Young permanent teeth injuries commonly occur in children aged 7–9 years and represent a frequent and significant condition in pediatric dental emergencies, accounting for approximately 50%–70% of permanent tooth injuries, with the upper central incisors being the most frequently affected. Dental trauma not only causes immediate local oral trauma but can also have adverse physical and psychological consequences for children (Laforgia et al. 2025). Avulsion, defined as the complete displacement of a tooth from its socket due to external force, is the most severe form of dental injury. It can result in the rupture of the periodontal ligament, loss of blood supply to the pulp tissue, and damage to the cementum, accounting for approximately 1%–16% of young permanent tooth injuries (Chappuis and von Arx 2005). The prognosis of replanted avulsed teeth is influenced by factors such as the time elapsed since avulsion, the storage medium, the patient's age, the stage of root development, and the replantation technique employed (Bourgeois et al. 2022).

The healing outcomes of replanted teeth include periodontal ligament healing, superficial resorption, inflammatory resorption, and RR. Periodontal ligament healing is the most desirable outcome, but due to various reasons, many patients are unable to receive timely replantation, resulting in suboptimal healing. Studies have shown that when avulsed teeth are stored in non‐physiological media for more than 60 min, the periodontal ligament cells are unlikely to survive, and delayed replantation is often necessary (Donaldson and Kinirons 2001). Delayed replantation rarely results in periodontal ligament healing or superficial resorption. RR or traumatic ankylosis often occurs following delayed replantation of avulsed teeth or severe intrusive injuries (Ideno et al. 2022). Mendes et al. through a meta‐analysis of the literature, reported that 51.0% of replanted teeth experienced RR, followed by inflammatory resorption (24.4%) and superficial resorption (13.3%) (Mendes et al. 2018). Studies indicate that RR typically occurs within 6 months to 1 year after trauma. In our study, the earliest occurrence of RR was observed in the first month after trauma (1 case), and the latest in the 24th month (1 case). The incidence of RR was 26.7% at 3 months, 70% at 6 months, consistent with previous literature reports (Hammarstrom and Lindskog 2010).

The pathogenesis of RR may be attributed to the drying, compression, and mechanical damage to the periodontal ligament during avulsion, leading to necrosis of periodontal ligament cells. The surface of the root is primarily covered by osteoclasts and osteoblasts. Osteoclasts cause resorption of the root surface, while osteoblasts generate new bone tissue, which gradually replaces the cementum and dentin of the root surface, resulting in RR (Ozturk Sheikholaemeh and Sengul 2025). The rate of RR varies with age, being slower in adults but significantly faster in adolescents due to their higher basal metabolic rate. Andreasen et al. (2007) found that young permanent teeth in the growth and development stage typically lose their roots completely within 3–7 years after the diagnosis of RR, leading to tooth loss. In our study, none of the 30 patients experienced tooth loss during the 2‐year follow‐up period, which may be attributed to the insufficient duration of follow‐up. As the follow‐up period progresses, the degree of RR is expected to gradually increase. Additionally, due to the low incidence of avulsion and the complexity of treatment, there is a certain rate of loss to follow‐up. Our study only included 30 patients, which may limit the generalizability of the results. Small sample size and short follow‐up time, existed some potential selection bias, limiting the robustness of multivariate analysis. Future studies with larger sample sizes or multicenter collaborations will further validate our findings.

The survival rate of periodontal ligament cells in avulsed teeth is closely related to the time elapsed since avulsion, the storage medium, and the stage of root development. Shorter avulsion times and shorter durations of dry storage result in a lower risk of RR. Conversely, longer avulsion times and storage in non‐physiological media significantly reduce the survival rate of periodontal ligament cells, increasing the likelihood of RR after replantation. In our study, all 30 cases involved delayed replantation, with avulsion times ranging from 2 to 48 h. Despite 19 cases being stored in physiological media, the prolonged avulsion time resulted in nearly complete necrosis of the periodontal ligament cells, leading to varying degrees of tooth sinking and RR during the 2‐year follow‐up period. Among the 30 cases, 15 patients exhibited progressive tooth sinking, with the degree of sinking gradually increasing. By the end of the 2‐year follow‐up, 2 patients had reached extreme ankylosis (Grade IV), and 3 had reached severe ankylosis (Grade III). The remaining 15 patients maintained mild tooth sinking without progression. When comparing the groups with progressive and stable tooth sinking, we found that the avulsion time was statistically significant, consistent with the findings of Lauridsen et al. (2020). In multivariate logistic regression analysis, the storage medium and root development stage did not show statistical significance, which may be due to the fact that all cases involved delayed replantation with avulsion times exceeding 60 min, thereby minimizing the influence of these factors.

Although RR ultimately leads to tooth loss, it generally extends the lifespan of the tooth compared to inflammatory resorption. The treatment of RR is complex, and currently, there is no ideal method to prevent its occurrence or progression in young permanent teeth. Decoronation is currently considered the best treatment option for young permanent teeth with RR. It effectively maintains the alveolar ridge height and width, and the eruption of adjacent teeth can promote vertical growth of the alveolar bone in the edentulous area, creating favorable conditions for future orthodontic treatment, dental implants, and permanent restoration (Araújo and Miranda 2023). Some scholars recommend performing decoronation before the peak growth period. If RR occurs during the early mixed dentition stage (7–10 years old), decoronation should be performed within 2 years. If it occurs during the late mixed dentition stage (> 10–12 years old), treatment plans should be tailored to the individual patient (Fouad et al. 2020). However, the treatment of RR in growing children is complicated by issues such as aesthetics, restoration of masticatory function, growth and development, space management, and orthodontic treatment. Our study highlights that longer avulsion times increase the risk of progressive tooth sinking and tooth loss. Therefore, it is crucial to enhance public awareness of emergency treatment for avulsion, optimize emergency trauma channels, reduce the incidence of delayed replantation, and prolong the lifespan of replanted teeth. This will be a key focus for pediatric stomatologists in the future.

## Author Contributions

**Gong Min** and **Qi Jianyan:** data collection, statistical analysis, initial manuscript drafting. **Cai Chenxing:** study design, supervision, critical revision. **Gao Suyu:** radiographic assessment, technical support.

## Ethics Statement

The study was approved by the Institutional Review Board of Nanjing Medical University (approval no.: PJ2019‐120‐001) and adheres to the Declaration of Helsinki. All patient data were anonymized, and no conflicts of interest exist among the authors. The manuscript is not under consideration for publication elsewhere.

## Conflicts of Interest

The authors declare no conflicts of interest.

## Data Availability

The data used to support the findings of this retrospective study on replacement resorption after replantation of avulsed young permanent teeth are not publicly available due to privacy and ethical constraints. The raw data include sensitive personal information (e.g., age, clinical records) and confidential clinical imaging data of patients, which are protected under the ethical approval guidelines of the Institutional Review Board of Nanjing Medical University (approval no.: PJ2019‐120‐001) to safeguard patient privacy. For researchers who meet the criteria for accessing confidential data (e.g., providing a valid research protocol and ethical approval from their respective institutions), requests to obtain the data can be directed to the corresponding author, Cai Chenxing, via the affiliated institution: Department of Pediatric Dentistry, The Affiliated Stomatological Hospital of Nanjing Medical University. All data requests will be reviewed to ensure compliance with privacy regulations and ethical standards before data access is granted.
